# Characteristics of Early Death in Patients With Localized Nasopharyngeal Cancer: A Population-Based SEER Analysis

**DOI:** 10.3389/fonc.2021.580220

**Published:** 2021-03-10

**Authors:** Haiyan Chen, Zhiheng Huang, Liubo Chen, Yanlin Li, Tiehong Zhao, Qichun Wei

**Affiliations:** ^1^ Department of Radiation Oncology, The Second Affiliated Hospital of Zhejiang University School of Medicine, Hangzhou, China; ^2^ Cancer Institute, Key Laboratory of Cancer Prevention and Intervention, Ministry of Education, The Second Affiliated Hospital of Zhejiang University School of Medicine, Hangzhou, China; ^3^ Department of Otorhinolaryngology, The Second Affiliated Hospital of Zhejiang University School of Medicine, Hangzhou, China; ^4^ Department of Colorectal Surgery and Oncology, Key Laboratory of Cancer Prevention and Intervention, Ministry of Education, The Second Affiliated Hospital, Zhejiang University School of Medicine, Hangzhou, China; ^5^ College of Science, Hangzhou Normal University, Hangzhou, China

**Keywords:** nasopharyngeal cancer, localized stage, early death, characteristics, Surveillance Epidemiology and End Results (SEER) program

## Abstract

Localized nasopharyngeal cancer (NPC) is a highly curable disease, but the prognosis of certain cases is still poor. Distinguishing patients with a poor outcome is necessary when developing therapeutic strategies. The aim of this study was to investigate the characteristics of early death (ED) among patients with localized NPC, and to identify independent predictors of ED. Patients diagnosed with localized NPC were included from the Surveillance, Epidemiology, and End Results dataset, and univariate and multivariate logistic regression analyses were performed to identify ED predictors. A total of 752 patients with localized NPC were enrolled, including 198 cases of ED and 480 long-term survivors. Older age, unmarried status, and white race were risk factors for ED, whereas diagnosis in the recent period and undifferentiated non-keratinizing histology type were protective factors. In addition, for older patients, women and those without radiation treatment, there was less ED for married patients than unmarried patients. In conclusion, this population-based study provides an overview of the characteristics of ED patients with localized NPC. Age, race, marital status, year of diagnosis and histology type are risk factors for ED. Moreover, married patients are at a significantly lower risk of ED. This protective effect is especially pronounced in older people, women and those without radiation treatment.

## Introduction

Nasopharyngeal cancer (NPC) is one of the common types of head and neck cancer (1). Unlike other head and neck malignancies, the geographical distribution pattern of NPC is very unique and uneven. In general, the incidence of NPC is lower than 1/100,000 in most areas, such as in Western countries, Latin America, and Japan (2, 3). However, in southern China, Southeast Asia, and in immigrants from these regions, the incidence rate can be as high as 20–30/100,000 in males and 10/100,000 in females (2, 4). The prognosis of NPC has improved significantly over the past decades because of advances in disease management, diagnostic imaging, radiotherapy technology, and broader application of systemic therapy (5). Despite these achievements, distant failure remains a key challenge and the burden of NPC will continuously increase especially in less developed countries, where it is estimated to increase 40%–50% by 2030 (1).

Due to its aggressive behavior and deep and complex anatomical location, NPC cases are diagnosed at a relatively late stage (6), and only 12%–20% of these patients present with early-stage NPC (5, 6). The American Joint Committee on Cancer (AJCC) staging system evolves rapidly and differs over the decades (6). Thus, it’s difficult to perform analysis using different editions of the staging system. In this study, we defined early-stage NPC as localized NPC, with cancer confined to the nasopharynx, or extension to oropharynx and/or nasal cavity without parapharyngeal involvement (7). Specifically, localized tumors were at stage I NPC according to the current 8^th^ edition AJCC cancer staging manual. The prognosis of localized NPC is favorable, and researchers have not determined outcome improvement with the addition of chemotherapy to radiotherapy (8). The main treatment modality for localized NPC is definite radiotherapy to nasopharynx and elective radiotherapy to neck (9, 10).

Localized NPC is a highly curable disease. The 5-year survival rate is approximately 87%–90% with conventional radiotherapy (6), and can be more than 95% with effective control *via* intensity-modulated radiotherapy (IMRT) (11). However, a very small proportion of patients have poor outcomes. These patients experience radiotherapy treatment failure, and might require much more intensive treatment at diagnosis, such as concurrent chemotherapy. Due to the rarity of these patients, there are currently very few data available regarding optimal management (8). It is critical to obtain more specific features of these localized NPC patients with poor prognosis.

In this study, we defined early death (ED) as cancer-specific death within 5 years of diagnosis of localized NPC. In addition to ED patients (EDPs), those who lived longer than 5 years were defined as long-term survivors (LTSs). Considering the small size of localized NPC samples, we used the largest publicly available cancer dataset, the Surveillance, Epidemiology, and End Results (SEER) dataset, to investigate the clinicopathological characteristics of EDP and LTS. In addition, we aimed to identify independent predictors of ED, and develop a nomogram to predict ED in localized NPC.

## Methods

### Data Source

We obtained internet access to the SEER Research Data 1975–2016 (www.seer.cancer.gov), which was released in April 2019, as based on the November 2018 submission (12). SEER is an authoritative source of information for cancer incidence and survival in the United States. This program contains data collected from 18 cancer registries, which cover 28% of the US population (13). It is the largest publicly available cancer dataset and is updated annually. SEER*Stat software (Surveillance Research Program, National Cancer Institute SEER*Stat software (www.seer.cancer.gov/seerstat) version 8.3.6.) was used. For selected patients, we collected data on demographics, primary tumor site, tumor morphology, disease stage at diagnosis, first course of treatment, and vital status at last contact. In this study, to investigate characteristics of EDPs, we only included patients diagnosed from 1975 to 2011 based upon SEER registries in 1975–2016, whose follow-up time was more than 5 years.

### Patient Selection and Diagnostic Codes

First, we included patients histologically diagnosed with NPC (Site recode ICD-O-3/WHO 2008: nasopharynx; Behavior recode: Malignant; Diagnostic Confirmation: Microscopically confirmed; Histologic type ICD-O-3: 8010, 8020, 8021, 8070, 8071, 8072, 8073, 8082). Then, we included patients with only one primary tumor (sequence number: one primary tumor only). Third, we only included those with localized disease (SEER historic stage A (1973–2015): localized, or SEER Combined Summary Stage 2000 (2004+): localized). These patients with localized NPC were included for demographic analyses. Considering the evolution of the AJCC staging system and the available AJCC staging information from 2004 (2004 AJCC 6^th^ edition and 2010 AJCC 7^th^ edition) (14, 15), we stratified the year of diagnosis into two time periods: 1975–2003 and 2004–2011. In addition, patients without active follow-up [SEER cause-specific death classification: Dead (missing/unknown COD), or N/A not first tumor] were excluded from survival analyses. EDP was defined as patient with cancer-specific death [SEER cause-specific death classification: Dead (attributable to this cancer dx)] within 5 years of diagnosis; those who lived longer than 5 years were defined as LTSs.

### Construction and Assessment of the Nomogram to Predict Early Death

Logistic regression analysis was performed to identify factors associated with ED. A nomogram was constructed based on the selected features from the logistic regression model. A receiving operator characteristic (ROC) curve and area under the curve (AUC) were used to assess the predictive accuracy. A calibration plot was constructed and the C-index was calculated to evaluate the calibration and discrimination of the nomogram. In addition, decision curve analysis (DCA) was quantified to determine the clinical utility of the model (16).

### Patients in the External Validation Cohort

In order to validate the performance of the prediction model, patients with localized NPC in the Second Affiliated Hospital of Zhejiang University School of Medicine (SAHZU) from 2011 to 2015 were consecutively enrolled. All the patients were histologically diagnosed as localized stage NPC without lymph node or distant organ metastasis. Twenty-two eligible patients were included in the SAHZU cohort. The characteristics for this external cohort were shown in **Table S1**. This project was approved by the Independent Ethics Committee of SAHZU and informed consent was obtained from all patients.

### Statistical Analysis

Pearson’s X^2^ test was conducted to investigate significant differences between two groups. Kaplan-Meier curves were plotted and the survival difference was analyzed by the log-rank test. Univariate and multivariate Cox regression analyses were conducted to generate odds ratios (ORs) with confidence intervals (CIs). SPSS statistics version 19.0 software (SPSS Inc, Chicago, United States) and R version 3.6.1 software (R Foundation for Statistical Computing, Vienna, Austria) were used to perform statistics. A P value of <0.05 was considered statistically significant. All tests were two sided, and 95% CIs are provided (17).

## Results

### Characteristics of Patients With Localized Nasopharyngeal Cancer

**Figure 1:**
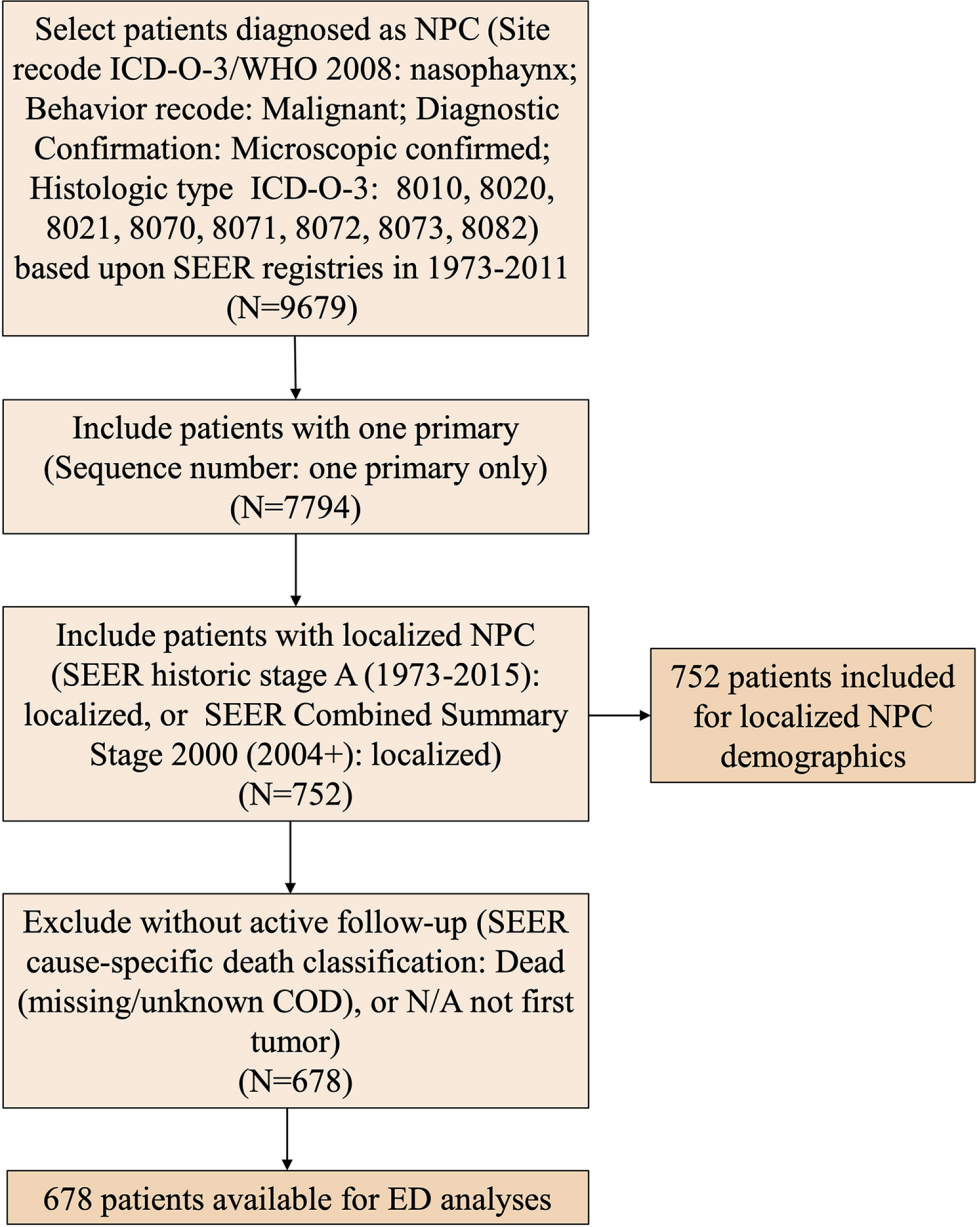
Flow chart of patient inclusion.

A total of 752 patients with localized NPC were assessed as eligible for inclusion in the study by using the patient selection algorithm described in the Methods section (**Figure 1**). The clinicopathological characteristics of these patients are shown in **Table 1**. Fourteen percent of these patients (*N* = 112) were younger than 40 years old, 47.21% of them (*N* = 355) were between 41 and 59 years old, and the rest (37.8%, *N* = 285) were more than 60 years old. Males (71.14%, N = 535) predominated. Regarding marital status, 67.69% (*N* = 509) of the patients were married, and 27.79% of them (*N* = 209) were unmarried, including divorced, separated, single, or widowed. Among these 752 patients, 40.69% (*N* = 306) were Asian or Pacific Islander, and 49.34% of them (*N* = 371) were white. In terms of the year of diagnosis, 66.62% of the patients were diagnosed from 1975 to 2003; 33.38% of them were diagnosed from 2004 to 2011. We also included histological type and found that 41.62% of the lesions were keratinizing squamous cell carcinomas, 14.63% were differentiated non-keratinizing carcinomas, and 25.00% were undifferentiated non-keratinizing carcinomas. Most of the patients (85.11%, *N* = 640) received radiation treatment. In addition, it is not surprising to find that overall survival (OS) (**Figure S1A**, *P* < 0.001) and cancer-specific survival (CSS) (**Figure S1B**, *P* < 0.001) improved statistically from 1975–2003 to 2004–2011.

**Table 1:** Clinical and pathological characteristics of included patients.

Characteristics	N	%
**Total**	752	100
**Age**		
≤40	112	14.89%
41–59	355	47.21%
≥60	285	37.90%
**Gender**		
Female	217	28.86%
Male	535	71.14%
**Marital Status**		
Married	509	67.69%
Divorce/Separate/Single/widowed	209	27.79%
Unknown	34	4.52%
**Race**		
Asian or Pacific Islander	306	40.69%
White	371	49.34%
Others	67	8.91%
Unknown	8	1.06%
**Year of Diagnosis**		
1975–2003	501	66.62%
2004–2011	251	33.38%
**Histology Type**		
Keratinizing squamous cell	313	41.62%
Differentiated non-keratinizing	110	14.63%
Undifferentiated non-keratinizing	188	25.00%
Others	141	18.75%
**Radiation Treatment**		
Yes	640	85.11%
No	34	4.52%
Unknown	70	9.31%

### Differences in Clinicopathological Characteristics Between Early Death Patients and Long-Term Survivors Groups

**Figure 2:**
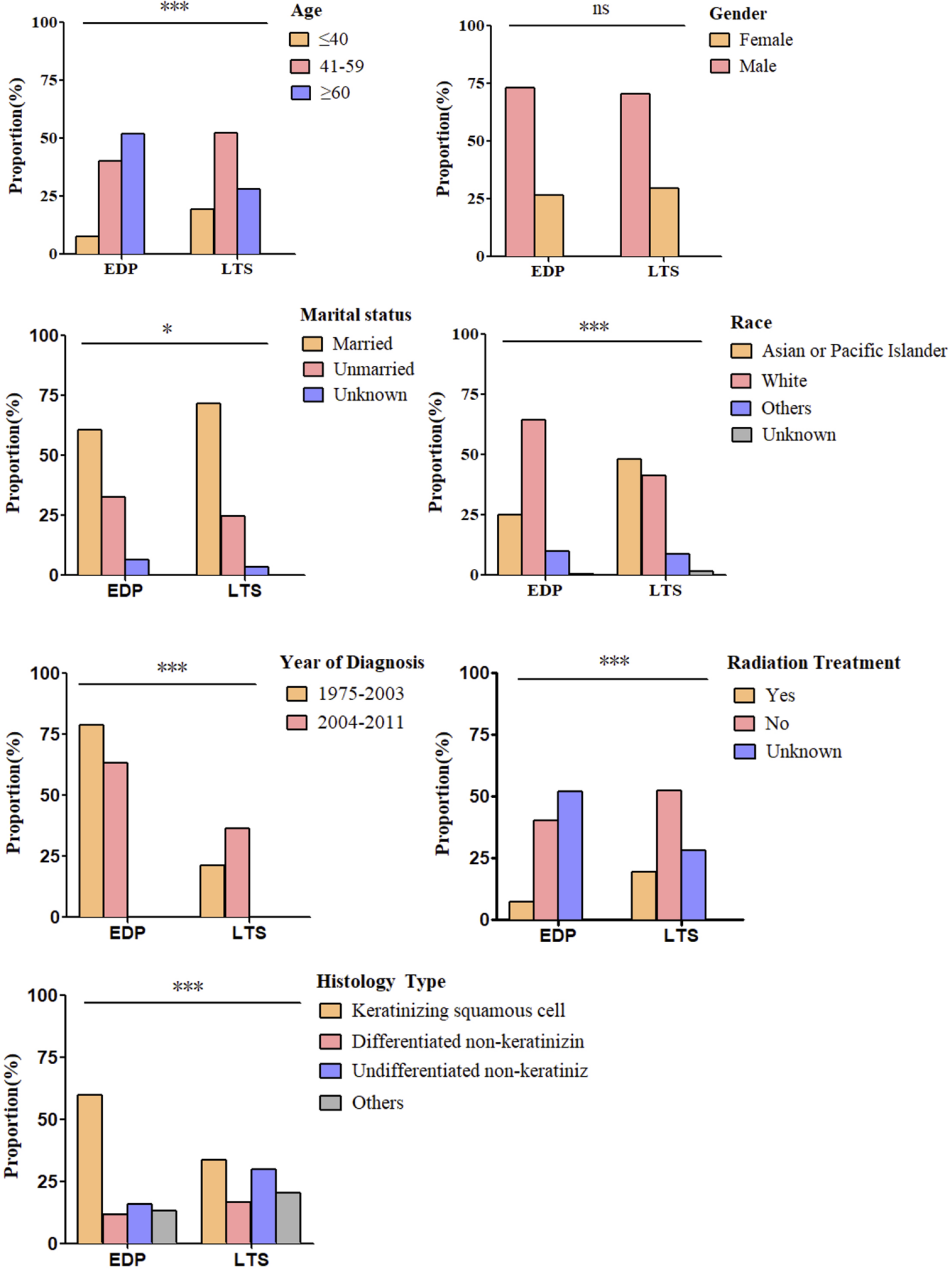
Differences in clinicopathological characteristics between EDP (*N* = 198) and LTS (*N* = 480) groups. Illustrated is the percentage of EDPs and LTSs for each included factor, including age (*P* < 0.001), sex (*P* = 0.26), marital status (*P* = 0.01), race (*P* < 0.001), year of diagnosis (*P* < 0.001), histology type (*P* < 0.001) and radiation treatment (*P* < 0.001). (ns, no significance. *P < 0.05, ***P < 0.001). The analysis was conducted using the Pearson X^2^ test.

By comparing EDPs and LTSs (**Table 2**, **Figure 2**), we found that there were a greater number of patients older than 60 (52.02% vs. 28.13%), and fewer younger than 40 (7.58% vs. 19.58%) among EDPs than LTSs (*P* < 0.001). Gender proportions did not statistically differ between the two groups. Regarding marital status, there were many fewer married patients (60.61%) in the EDP group than in the LTS group (71.67%) (*P* = 0.01). In addition, fewer Asian or Pacific Islander (25.25% vs. 48.13%) and more white (64.65% vs. 41.46%) patients were found among the EDP than LTS group (*P* < 0.001). Regarding the year of diagnosis, EDPs were diagnosed earlier than LTSs (*P* < 0.001). Regarding histological type, keratinizing squamous cell carcinoma was much more common in EDPs (59.60%) than in LTSs (34.54%), and the percentages of differentiated non-keratinizing carcinoma (11.62% vs. 16.46%) and undifferentiated non-keratinizing carcinoma were lower (15.66% vs. 29.79%) (*P* < 0.001) among EDPs. Last, there was a smaller population of patients receiving radiation treatment in the EDP group (80.30% vs. 88.96%) (*P* < 0.001).

**Table 2:** Differences of clinicopathological characteristics between EDP and LTS.

	EDP						LTS		P Value
	<1 Year		2–5 Year		Total				
**Total**	57	100.00%	141	100.00%	198	100.00%	480	100.00%	
**Age**									<0.001^╊^
≤40	1	1.75%	14	9.93%	15	7.58%	94	19.58%	
41–59	19	33.33%	61	43.26%	80	40.40%	251	52.29%	
≥60	37	64.91%	66	46.81%	103	52.02%	135	28.13%	
**Gender**									0.26^╊^
Female	17	29.82%	36	25.53%	53	26.77%	142	29.58%	
Male	40	70.18%	105	74.47%	145	73.23%	338	70.42%	
**Marital Status**									0.01^╊^
Married	34	59.65%	86	60.99%	120	60.61%	344	71.67%	
Unmarried	19	33.33%	46	32.62%	65	32.83%	119	24.79%	
Unknown	4	7.02%	9	6.38%	13	6.57%	17	3.54%	
**Race**									<0.001^╊^
Asian or Pacific Islander	10	17.54%	40	28.37%	50	25.25%	231	48.13%	
White	43	75.44%	85	60.28%	128	64.65%	199	41.46%	
Others	4	7.02%	16	11.35%	20	10.10%	42	8.75%	
Unknown	0	0.00%	1	0.71%	1	0.51%	8	1.67%	
**Year of Diagnosis**									<0.001^╊^
1975–2003	44	77.19%	112	79.43%	156	78.79%	304	63.33%	
2004–2011	13	22.81%	29	20.57%	42	21.21%	176	36.67%	
**Histology Type**									<0.001^╊^
Keratinizing squamous cell	36	63.16%	82	58.16%	118	59.60%	161	33.54%	
Differentiated non-keratinizing	5	8.77%	18	12.77%	23	11.62%	79	16.46%	
Undifferentiated non-keratinizing	7	12.28%	24	17.02%	31	15.66%	143	29.79%	
Others	9	15.79%	17	12.06%	26	13.13%	97	20.21%	
**Radiation Treatment**									<0.001^╊^
Yes	36	63.16%	123	87.23%	159	80.30%	427	88.96%	
No	3	5.26%	5	3.55%	8	4.04%	26	5.42%	
Unknown	18	31.58%	13	9.22%	31	15.66%	27	5.63%	

^╊^Pearson x^2^ test.

### Identification of Early Death Predictors in Localized Nasopharyngeal Cancer

**Figure 3:**
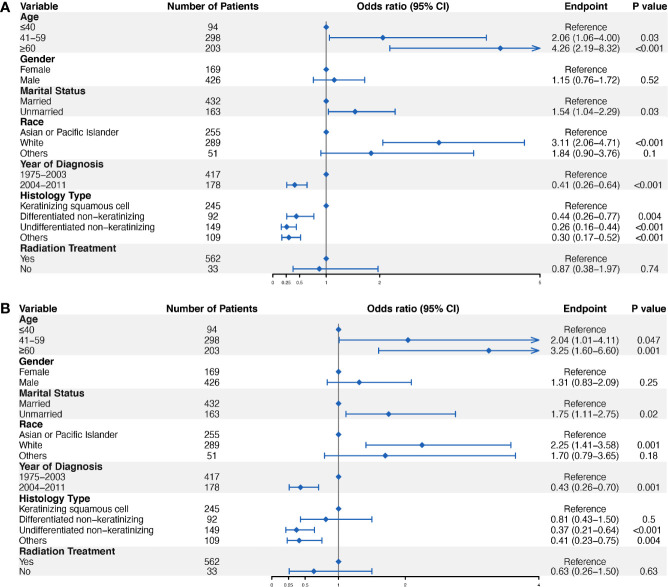
Logistic regression analyses to identify ED predictors in localized NPC. Univariate logistic regression analysis **(A)** and multivariate logistic regression analysis **(B)** were conducted to identify factors significantly associated with ED.

We performed univariate and multivariate logistic regression analyses to identify predictors of ED for localized NPC. The results showed that patients at an older age (41–59 years old, OR = 2.04, 95% CI = 1.01–4.11, *P* = 0.047; ≥60 years old, OR = 3.25, 95% CI = 1.60–6.60, *P* = 0.001), with an unmarried status (OR = 1.75, 95% CI = 1.11–2.75, *P* = 0.02), and white (OR = 2.25, 95% CI = 1.41–3.58, *P* = 0.001) were more likely to have ED events (**Figure 3**). In contrast, patients diagnosed in 2004–2011(OR = 0.43, 95% CI = 0.26–0.70, *P* = 0.001), with the undifferentiated non-keratinizing histology type (OR = 0.37, 95% CI = 0.21–0.64, *P* < 0.001) were less likely to have ED events (**Figure 3**). Furthermore, we performed subgroup analysis in patients diagnosed from 2004 to 2011, and the results confirmed the role of marital status in the prediction of ED. Specifically, unmarried patients were more likely to have ED for localized NPC diagnosed from 2004 to 2011 (OR = 2.80, 95% CI = 1.07–7.35, *P* = 0.04) (**Figure S2**).

### Construction and Assessment of the Nomogram

**Figure 4:**
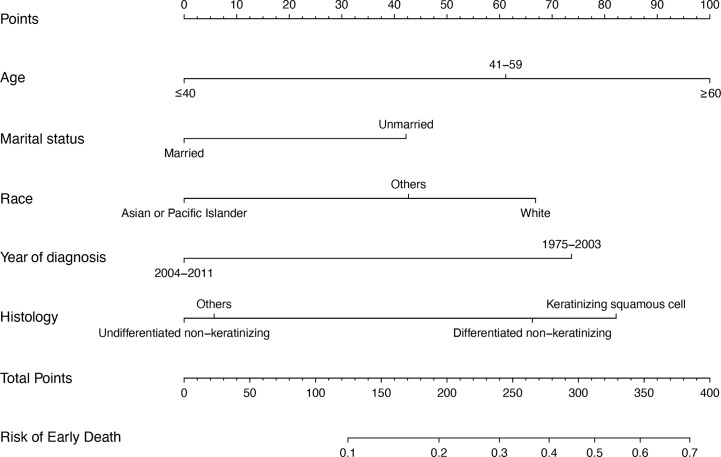
The nomogram model for predicting ED probabilities of patients with localized NPC. For example, there is one patient with localized NPC older than 60 (100 points), with married status (0 point), the race of Asian (0 point). He is diagnosed in 2010 (0 point), and the histology type is differentiated non-keratinizing tumor (60 points). These five values summed to 160 points. Therefore, we determined that the risk of ED was 0.1–0.2.

**Figure 5:**
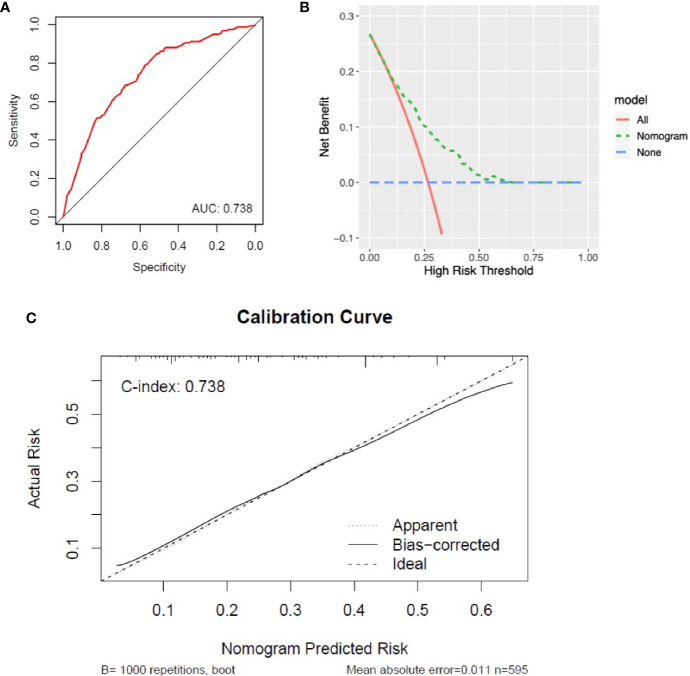
Assessment of the performance of nomogram in the SEER cohort. **(A)** The AUC was 0.73 (95% CI: 0.69–0.78) for ED in patients with localized NPC. **(B)** DCA for the nomogram in predicting ED. **(C)** Calibration curve of nomogram-predicted ED risk and actual risk.

A nomogram was generated to assign the ED probability of an individual based on selected features in the multivariate logistic model, including age, gender, marital status, year of diagnosis, and histology type (**Figure 4**). The AUC for the nomogram was 0.73 (95% CI: 0.69–0.78) (**Figure 5A**), and DCA results indicated that the nomogram model had a greater net benefit compared to “treat-all” and “treat-none” strategies when the risk threshold ranged between approximately from 0.5 to 0.8, indicating its good clinical utility (**Figure 5B**). In addition, the calibration plot matched well with the ideal 45-degree line, implying good consistency between the model predictions and actual observations for ED (**Figure 5C**). Besides, we used an independent cohort from the SAHZU cancer center to perform external validation on this prediction model. Limited by the low incidence rate of localized NPC, there were 22 patients included, among which 3 patients had ED. The AUC was 0.68 (95% CI 0.18–1.00) in the SAHZU validation cohort (**Figure S3A**), and the nomogram model demonstrated good clinical utility (**Figure S3B**).

### Marital Status Was an Independent Early Death Factor for Localized Nasopharyngeal Cancer

From the multivariate analyses, we found that marital status is a crucial risk factor for ED in localized NPC. To further understand the association between marital status and ED, we performed stratified analyses to compare ED between patients of different marital statuses (**Table 3**). The results of stratified analyses based on age showed that for patients older than 60, there was a lower percentage of ED among married patients than unmarried patients (32.59% vs. 50.00%) (*P* = 0.01). Similarly, for female patients (17.17% vs. 35.71%) (*P* = 0.005), those diagnosed from 1975 to 2003 (28.90% vs. 38.53%) (*P* = 0.04) and those without radiation treatment (12.50% vs. 55.56%) (*P* = 0.02), a statistically lower ED rate was found in the married group than in the unmarried group.

**Table 3:** Marital Status and ED in Localized NPC.

Characteristics	Total	Married				Unmarried				P Value
		Total		ED		Total		ED		
		N	%	N	%	N	%	N	%	
**Total**	595	427		105		159		54		
**Age**										
≤40	94	55	58.51%	6	10.91%	39	41.49%	6	15.38%	0.37^╊^
41–59	298	242	81.21%	55	22.73%	56	18.79%	14	25.00%	0.42^╊^
≥60	203	135	66.50%	44	32.59%	68	33.50%	34	50.00%	0.01^╊^
**Gender**										
Female	169	99	58.58%	17	17.17%	70	41.42%	25	35.71%	0.005^╊^
Male	426	333	78.17%	88	26.43%	93	21.83%	29	31.18%	0.22^╊^
**Race**										
Asian or Pacific Islander	255	194	76.08%	28	14.43%	61	23.92%	12	19.67%	0.22^╊^
White	289	209	72.32%	72	34.45%	80	27.68%	34	42.50%	0.13^╊^
Others	51	29	56.86%	5	17.24%	22	43.14%	8	36.36%	0.11^╊^
**Year of Diagnosis**										
1975–2003	417	308	73.86%	89	28.90%	109	26.14%	42	38.53%	0.04^╊^
2004–2011	178	124	69.66%	16	12.90%	54	30.34%	12	22.22%	0.09^╊^
**Histology Type**										
Keratinizing squamous cell	245	179	73.06%	67	37.43%	66	26.94%	31	46.97%	0.11^╊^
Differentiated non-keratinizing	92	65	70.65%	14	21.54%	27	29.35%	7	25.93%	0.42^╊^
Undifferentiated non-keratinizing	149	113	75.84%	15	13.27%	36	24.16%	7	19.44%	0.26^╊^
Others	109	75	68.81%	9	12.00%	34	31.19%	9	26.47%	0.06^╊^
**Radiation Treatment**										
Yes	562	408	72.60%	102	25.00%	154	27.40%	49	31.82%	0.07^╊^
No	33	24	72.73%	3	12.50%	9	27.27%	5	55.56%	0.02^╊^

^╊^Pearson x^2^ test.

## Discussion

Most NPC patients are diagnosed at a locally advanced stage, and only approximately 12% of patients present with localized NPC (5). Therefore, it is very difficult to perform demographic or survival analyses based on single institution data because of the small sample size. Therefore, we investigated the features of patients with localized NPC by using the SEER Program, which is the largest publicly available cancer dataset (12). To the best of our knowledge, the present research is the largest and most up-to date population-based study of localized NPC, consisting of 752 eligible patients for demographic analyses. Further survival analyses showed that the prognosis for most of patients with localized NPC was good and improved dramatically over time. According to the latest NCCN clinical practice guidelines, patients with early-stage NPC are recommended to receive radiotherapy alone (10). However, some patients have a poor prognosis. For these patients, efforts should be made to deliver more precise and individualized therapy (6). For example, they might receive much more intensive treatment, such as radiotherapy and concurrent chemotherapy, or be followed up much more frequently to achieve good survival outcomes.

Overall, selecting patients with localized NPC and a poor prognosis has become a major issue. In this study, multivariate logistic regression analyses showed that patients at an older age, with an unmarried status and white were more likely to have ED but that those diagnosed during 2004–2011 and with the undifferentiated nonkeratinizing histology type were less likely to have ED. These parameters are easily accessible from routine clinical work. Based on these results, we constructed a nomogram, a simple and visual tool for prediction (18), to predict ED in localized NPC. The performance of the nomogram was verified by calibration, discrimination and its clinical utility, as indicated by the calibration plot, AUC, C-index, and DCA result in both SEER and SAHZU cohort. This model will help distinguish high- and low-risk EDPs in localized NPC. For example, one patient with localized NPC was older than 60 (100 points), married (0 points), and Asian (0 points). He was diagnosed in 2010 (0 points), and the histology type was a differentiated non-keratinizing tumor (60 points). These five values summed to 160 points. Therefore, we determined that the risk of ED was 0.1–0.2 (19). Of course, whether high-risk patients need much more intensive treatment and surveillance strategies requires further study.

It has been shown that age is a strong independent prognostic factor for NPC (20). In our study, people over 40 had a higher risk of ED than those under 40. Specifically, the risk of ED is the highest in patients aged >60, followed by patients aged 40–60. Regarding race, NPC is a relatively rare cancer type in the white population, but the prognosis is poorer than that in Asian people (21). The reason might be because of differences in histology type. It has been reported that the histological type of most white NPC patients is keratinizing squamous cell carcinoma. Differentiated non-keratinizing and undifferentiated non-keratinizing types are much more common in Asian patients, and those with differentiated non-keratinizing and undifferentiated non-keratinizing histology types had the best survival. Similarly, we conclude that race and histology type are risk factors for ED in patients with localized NPC.

It is interesting to find that marital status is a protective factor for ED. Spousal support plays an important role in the active surveillance and care of patients, and married people have a significantly higher probability of receiving definitive treatment, thus contributing to better survival (22). In contrast, unmarried patients are more likely to experience distant metastasis, undertreatment, and death resulting from their cancer (23). In addition to head and neck cancer (22), marital status has been implicated as a prognostic factor in pancreatic ductal adenocarcinoma (24), colorectal cancer (25), bladder cancer (26), ovarian cancer (27), and other cancer types (23). Our stratified analyses of marital status and ED suggest that marriage significantly protects older people (>60) and women from ED. This implies that in localized NPC, older and female patients and patients without radiation treatment need more psychosocial support, which can be partially fulfilled by the support system provided by marriage.

In addition to the factors included in our study, recent studies have demonstrated that hematological biomarkers, which are easy to access for clinicians, are associated with a poor prognosis in NPC (28). For example, Chan et al. (29) reported that the level of plasma EBV DNA correlated significantly with locoregional failure, distant metastasis, and death in NPC patients after chemoradiation therapy. Inflammation-based factors, such as the C-reactive protein/albumin ratio, platelet/lymphocyte ratio, and neutrophil/lymphocyte ratio, have been reported to correlate with survival in NPC (30). In addition, high-density lipoprotein cholesterol, apolipoprotein A-1, albumin, and hemoglobin are prognostic factors (28). Li et al. (28) proposed a nomogram incorporating hematological risk factors and clinical characteristics, showing higher predictive power than the classical AJCC staging system. However, whether these hematological biomarkers can predict ED in localized NPC is unknown and requires further study with the inclusion of many more potential factors.

IMRT significantly improves survival compared with non-IMRT, enhancing quality of life and lowering radiation toxicity (31). However, in this SEER-based observational study, we were unable to obtain data regarding radiation technology. We also lack information on adjuvant chemotherapy, comorbidities, complications, and molecular features of the patients, which are all strongly associated with prognosis. This is one of the limitations of this study. Moreover, given that the AJCC staging system has evolved and differed over the decades (32, 33), we used the SEER historical staging classification instead. For cases diagnosed from 2004 to 2011, it will be better to divided them into T1N0M0 or T2aN0M0 tumors according to the AJCC 6^th^ edition staging system, to minimize heterogeneity. In addition, because of the relatively low incidence rate of localized NPC, the sample size of external validation cohort based on single institution is small, which lowered the reliability of our findings and limited validation of the nomogram. A prospective multicenter population-based with a large sample size is warranted to validate the results in our present study. Finally, the logistic regression model assumes that the outcome is a linear combination of covariates, but the patient data are diverse and complex and cannot be generally considered linear. A machine learning-based model is required in the future to detect the relationships from complex datasets.

In conclusion, in this population-based study, we investigated the characteristics of localized NPC and predictors of ED according to SEER data. Older age, unmarried status and white race were risk factors for ED, whereas diagnosis in the recent period and undifferentiated non-keratinizing histology were protective factors. Moreover, married patients were at a significantly lower risk of ED. This protective effect was especially pronounced in older people, women and those without radiation treatment.

## Data Availability Statement

The original contributions presented in the study are included in the article/**Supplementary Material**. Further inquiries can be directed to the corresponding author.

## Ethics Statement

The studies involving human participants were reviewed and approved by Independent Ethics Committee of Second Affiliated Hospital of Zhejiang University School of Medicine. The patients/participants provided their written informed consent to participate in this study.

## Author Contributions

QW designed the study. HC and ZH collected and analyzed the data. YL and TZ helped to perform the statistical analysis. HC and LC wrote the manuscript. All authors contributed to the article and approved the submitted version.

## Funding

This work was partly supported by grants from the National Natural Science Foundation of China (82073332), China Postdoctoral Science Foundation (519000-X91919), Zhejiang Provincial Natural Science Foundation of China (LQ21H160035), CSCO-Roche research funding (Y-Roche2019/2-0088), and the Medical Health Science and Technology Project of the Health Commission of Zhejiang province (2020381184).

## Conflict of Interest

The authors declare that the research was conducted in the absence of any commercial or financial relationships that could be construed as a potential conflict of interest.

## Supplementary Material

Supplementary Figure 1 Overall survival **(A)** and cancer-specific survival **(B)** of localized NPC patients stratified by year of diagnosis.

Supplementary Figure 2 Univariate logistic regression analysis **(A)** and multivariate logistic regression analysis **(B)** was used to determine predictive factors for ED in localized NP patients diagnosed from 2004 to 2011.

Supplementary Figure 3 Assessment of the performance of nomogram in the SAHZU cohort. **(A)** The AUC was 0.68 (95% CI 0.18-1.00) in the SAHZU validation cohort. **(B)** DCA for the nomogram in predicting ED in SAHZU cohort.
