# Supplementary figures and images for: Characteristics of Early Death in Patients With Localized Nasopharyngeal Cancer: A Population-Based SEER Analysis
Source: Front Oncol. 2021 Mar 10;11:580220. doi: 10.3389/fonc.2021.580220 (PMC8006381; doi:10.3389/fonc.2021.580220)

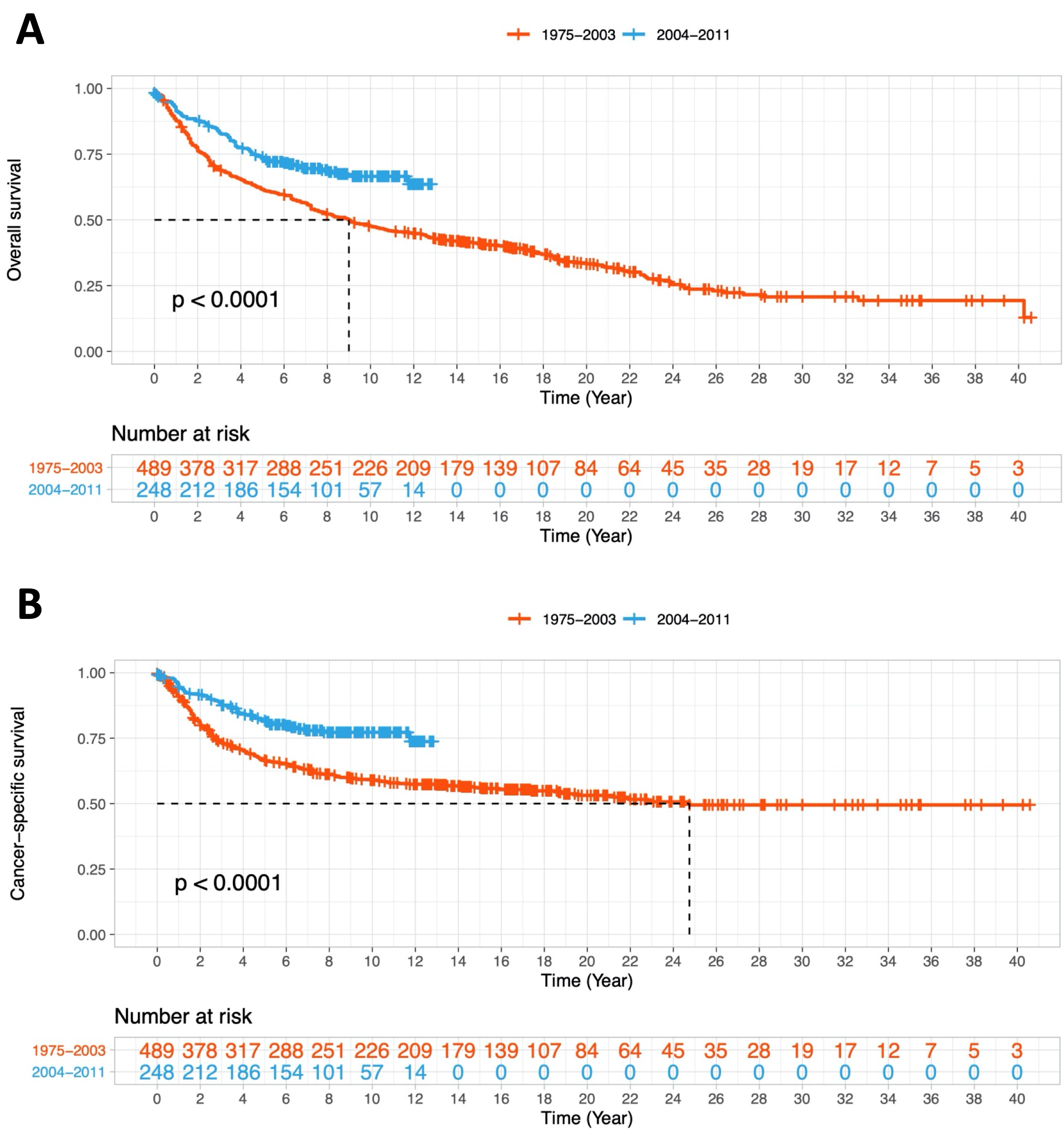

Supplement: Supplementary Figure 1 — Overall survival (A) and cancer-specific survival (B) of localized NPC patients stratified by year of diagnosis. [file Image_1.jpeg]

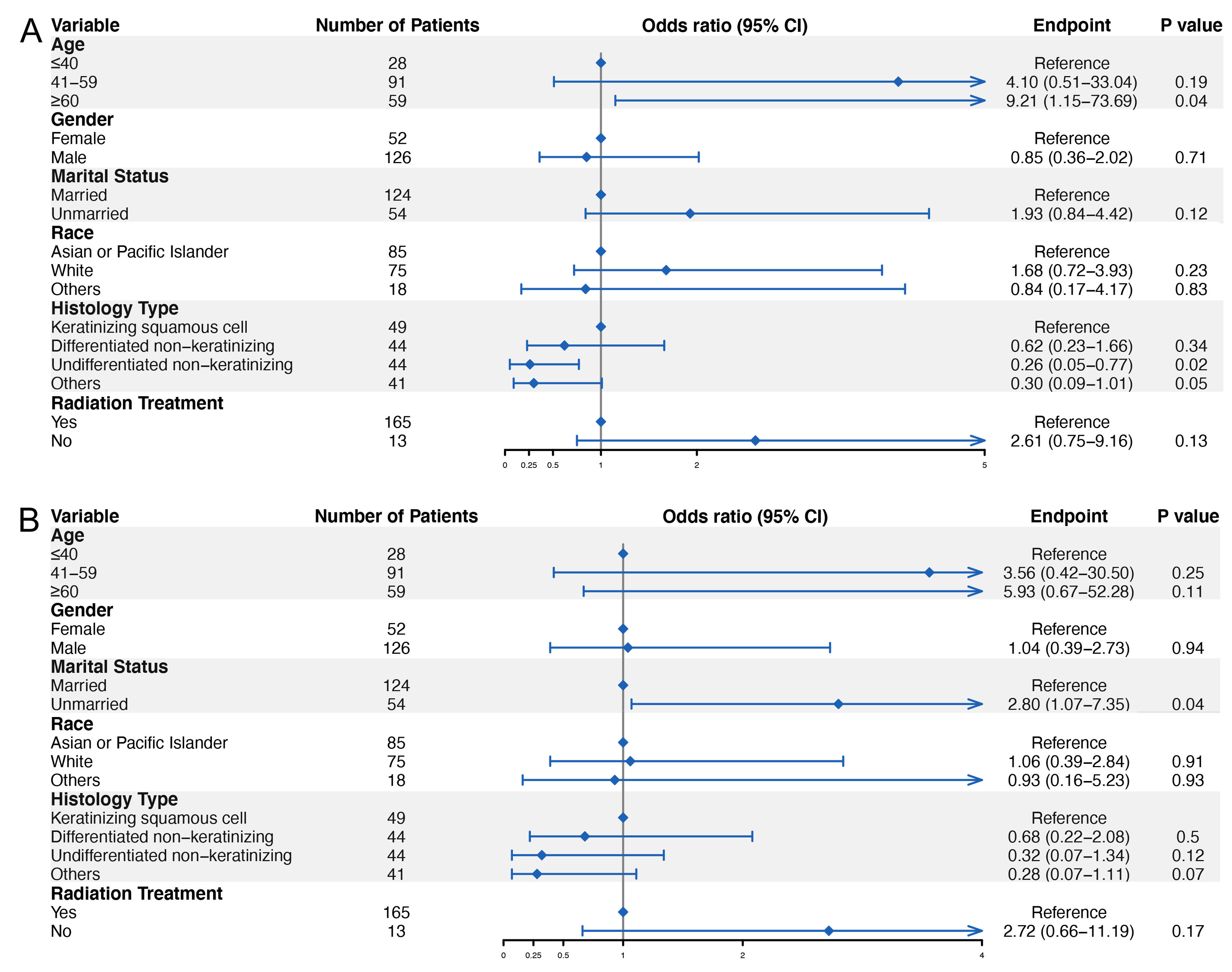

Supplement: Supplementary Figure 2 — Univariate logistic regression analysis (A) and multivariate logistic regression analysis (B) was used to determine predictive factors for ED in localized NP patients diagnosed from 2004 to 2011. [file Image_2.tif]

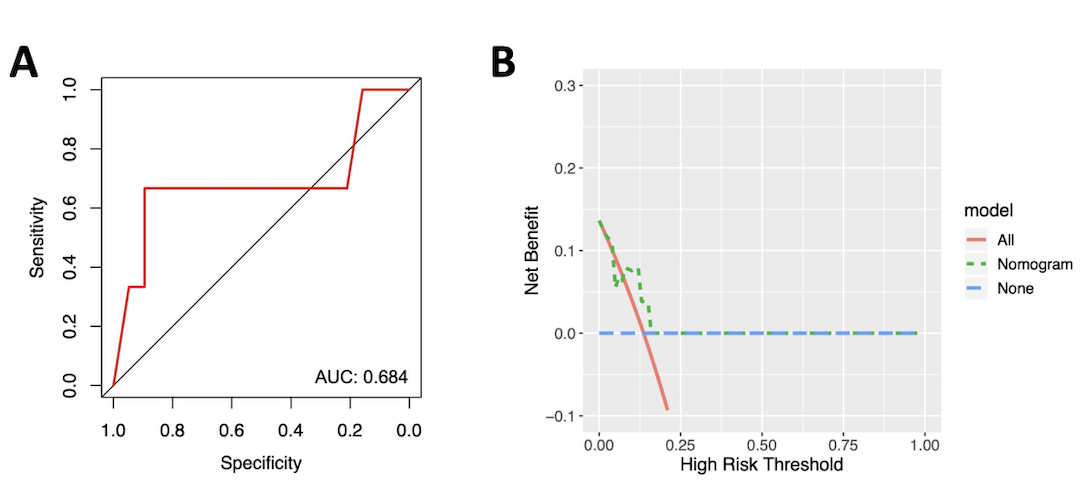

Supplement: Supplementary Figure 3 — Assessment of the performance of nomogram in the SAHZU cohort. (A) The AUC was 0.68 (95% CI 0.18-1.00) in the SAHZU validation cohort. (B) DCA for the nomogram in predicting ED in SAHZU cohort. [file Image_3.png]
