# Supplementary material for: Characteristics of Early Death in Patients With Localized Nasopharyngeal Cancer: A Population-Based SEER Analysis
Source: Front Oncol. 2021 Mar 10;11:580220. doi: 10.3389/fonc.2021.580220 (PMC8006381; doi:10.3389/fonc.2021.580220)
Supplement: Supplementary file 4 [file Table_1.docx]

| **Table S1. Characteristics of the SAHZU cohort** | | |
| --- | --- | --- |
| **Characteristics** | **N** | **%** |
| **Total** | 22 | 100 |
| **Age** |  |  |
| ≤40 | 5 | 22.73% |
| 41-59 | 9 | 40.91% |
| ≥60 | 8 | 36.36% |
| **Gender** |  |  |
| Female | 8 | 36.36% |
| Male | 14 | 63.64% |
| **Marital Status** |  |  |
| Married | 19 | 86.36% |
| Divorce/Separate/Single/widowed | 3 | 13.64% |
| **Race** |  |  |
| Asian or Pacific Islander | 22 | 100.00% |
| White | 0 | 0.00% |
| Others | 0 | 0.00% |
| **Year of Diagnosis** |  |  |
| 1975-2003 | 0 | 0.00% |
| ≥2004* | 22 | 100.00% |
| **Histology Type** |  |  |
| Keratinizing squamous cell | 1 | 4.55% |
| Differentiated non-keratinizing | 3 | 13.64% |
| Undifferentiated non-keratinizing | 18 | 81.82% |
| Others | 0 | 0.00% |
| **Early Death** |  |  |
| No | 19 | 86.36% |
| Yes | 3 | 13.64% |

* These patients were diagnosed from 2011 to 2015. When performing validation, they were grouped into recently diagnosed subgroup (2004-2011).
